# Efficient management of new patient referrals to a breast service: the safe introduction of an advanced nurse practitioner-led telephone breast pain service

**DOI:** 10.1308/rcsann.2023.0056

**Published:** 2023-08-29

**Authors:** KS Ellis, CE Robinson, R Foster, H Fatayer, A Gandhi

**Affiliations:** ^1^ Manchester University NHS Foundation Trust, UK; ^2^ Manchester Academic Health Sciences Centre, University of Manchester, UK

**Keywords:** Mastalgia, Breast pain, Telephone assessment, Nurse-led, Referrals, Advanced nurse practitioner

## Abstract

**Introduction:**

There has been an almost 100% increase in referrals to breast cancer diagnostic clinics in the past decade. Breaching of the two-week cancer referral target is now commonplace, potentially delaying diagnoses of breast malignancy in many women. Almost one in five of these referrals are women with mastalgia, not a symptom linked to breast cancer. The objective of the study was the safe introduction of an advanced nurse practitioner-led telephone service for women with mastalgia to improve the service for women and create capacity for those with “red flag” breast symptoms.

**Methods:**

Referrals to clinic were triaged, women with mastalgia only were directed to a telephone-based assessment clinic and symptoms evaluated using a multidisciplinary created proforma.

**Results:**

Within 23 months, 1,427 women were assessed in the breast pain telephone assessment clinic: 863 (61%) were aged over 40 and 564 (39%) aged under 40. A total of 1,238 underwent telephone assessment. Reassurance and discharge only was needed for 365 (26%). The aetiology of pain was identified as musculoskeletal in 1,104/1,238 (89%) of patients, with only 39/1,238 (3.2%) identified as having true breast pain. Additional symptoms were mentioned by 264 women (18%) during the consultation; all immediately redirected back to a diagnostic clinic. Mammography was undertaken in 609 women (43%). Seven women (0.6%) were diagnosed with a breast malignancy. Patient survey indicated that 93% of patients were satisfied with the care received and 97% said they would recommend the service to a family member or friend.

**Conclusions:**

Although face-to-face assessments for breast pain remain the standard practice in many breast units, data indicating the safety of a telephone assessment clinic, along with high levels of patient satisfaction, question whether services can be delivered differently.

## Introduction

Mastalgia is a common complaint among women of all ages and is suggested to be experienced by around 70% of women at some point in their life.^1^ The aetiology of mastalgia maybe divided into pain originating from the breast, musculoskeletal pain (perceived to be originating from the breast but actually arising from the chest wall adjacent to the breast) or referred pain from other aetiology. Pain originating from the breast may be found to be cyclical with symptoms influenced by the patient’s menstrual cycle, or non-cyclical where breast pain symptoms are not influenced by the menstrual cycle or are noted in postmenopausal women.^1^ Musculoskeletal breast pain is considered the most common type of pain referred into a breast clinic.^2^

The past 10 years has seen an almost 100% increase in numbers of patient referrals to breast clinics,^3^ with referrals for mastalgia alone accounting for anywhere between 13% and 50%^4,5^ possibly because mastalgia is associated with a concern that it may represent a symptom of breast cancer.^6,7^ However, mastalgia as a solitary symptom is very unlikely to be linked with a breast malignancy,^8^ and a recent comprehensive large prospective study of almost 11,000 symptomatic breast clinic referrals suggested a breast cancer incidence of 0.4% in women with a solitary symptom of mastalgia.^9^

Challenges to previous working practices generated by the COVID-19 pandemic and consequent increased pressures on health service capacity have made prioritising urgent cancer-related outpatient reviews crucial for women with clinically worrying red flag symptoms such as breast lumps or breast distortion. To achieve this, while maintaining a safe and effective pathway for the assessment of women with mastalgia, an advanced nurse practitioner (ANP)-led telephone clinic was established with strict referral criteria and outcome measures.

The aims of this study were to scrutinise the safety of this clinic, report on patient outcomes and determine levels of patients’ satisfaction with the service.

## Methods

Following detailed multidisciplinary discussions, a robust pathway was developed to provide a telephone-based assessment service for patients with breast pain as a solitary symptom outside a breast cancer diagnostic clinic. The urgency of the COVID-19 pandemic enabled rapid organisation and implementation of this initiative in May 2020.

All referrals to the specialist breast cancer diagnostic clinic were triaged, enabling each referral to be categorised into one of two groups: women with mastalgia as the sole reason for referral (Group 1); and women with all other breast symptoms such as breast lumps, nipple symptoms, breast skin changes or any other breast symptomatology (Group 2).

No distinction was made between women with cyclical or non-cyclical mastalgia for inclusion into Group 1. Women with mastalgia in addition to any other breast symptom (e.g. breast lump, nipple discharge) were categorised into Group 2. The presence or absence of these other breast symptoms was defined by the referral document from the referring clinician and verified by the patient on clinical review. Patients with breast implants were included into Group 2 because mastalgia in this group could potentially be representative of capsular contracture and/or implant rupture, which would be difficult to assess and diagnose outside a face-to-face clinic. Patients previously treated for breast cancer were also included in Group 2.

Daily triaging enabled women with mastalgia as a solitary symptom (Group 1) to be transferred from the breast cancer diagnostic clinic pathway to the specifically designed telephone mastalgia clinic pathway. All other women (Group 2) continued with a standard diagnostic clinic face-to-face assessment.

Telephone-based mastalgia assessment clinics were delivered concurrently by two ANPs, with each patient allocated a 15-minute slot and patients being informed about the appointment by letter. A proforma completed during the consultation included questions about symptoms: type of pain, duration, exacerbating/relieving factors, family history, any other symptoms, most recent mammogram (if any) and menstrual status, with all outcomes recorded. Patients failing to answer the telephone after two attempts for an allocated appointment time were offered one further appointment.

Patients identified during the telephone consultation as having additional breast symptoms (i.e. not mastalgia as the solitary symptom) were redirected to a one-stop clinic for a face-to-face assessment.

Patients over the age of 40 were referred for bilateral mammography unless they had undergone this investigation in the previous 6 months. Mammograms were scheduled outwith any one-stop breast cancer diagnostic clinic. If mammographic anomalies were identified by specialist breast radiologists, patients were informed of the anomaly by telephone consultation and were invited to a breast diagnostic clinic within 7 days where they underwent clinical examination and further investigations as clinically indicated.

The aetiology of patients’ symptoms was collected prospectively. Musculoskeletal pain was classified using an assessment tool created in a face-to-face environment and was defined as being non-cyclical (occurring outside the premenstrual menstrual phase or in postmenopausal women). Cyclical mastalgia was defined as pain occurring in premenopausal women, with severity of symptoms reported as being chronologically linked to the woman’s menstrual cycle.

Before commencing the telephone-based service, an equality impact assessment was undertaken to mitigate against those deemed to be at any unnecessary disadvantage from a telephone consultation compared with a face-to-face assessment. Patients who appeared to be at a disadvantage could include, but were not limited to, patients requiring an interpreter or those with known hearing difficulties or speech problems. These patients were triaged into Group 2 and seen in a traditional face-to-face breast clinic appointment.

Immediately after the consultation, patients in Group 1 were sent an SMS text message questionnaire. Questions included satisfaction about the telephone assessment, the timing of the appointment, whether the patient believed that a telephone consultation saved them time and whether they would refer the service to family and friends.

To investigate whether the telephone mastalgia clinic pathway was successful in providing a resolution to the women’s concerns about their symptoms, we examined how many women assessed in the telephone clinic returned with further related or unrelated breast symptoms within 12 months of their telephone appointment. Data were retrieved from the trust information department of all the patients assessed via the telephone mastalgia clinic who, within 12 months of this assessment, had subsequently been re-referred to the breast unit. For each episode, it was ascertained why the patient had returned, and the outcome of the consultation.

## Results

Between May 2020 and April 2022, a total of 16,722 patients were referred to the South Manchester Breast Clinic of whom 1,427 (8.5%) were referred with a diagnosis of mastalgia only.

Of these 1,427 women, 188 (13%) did not engage with the allocated telephone appointment. Thus, 1,238 patients (87%) with a median age of 44 years (range 17–95) underwent telephone consultation for symptoms of mastalgia alone. There were 390 patients (32%) who fell within the current National Health Service (NHS) breast screening programme screening age range of 50–70 years; 705 (57%) were younger than 50 years and 143 (12%) were older than 70 years. The onset of symptoms ranged from several weeks to several years.

A total of 974 patients (79%) avoided a one-stop clinic appointment, whereas 264 (21%) were referred onwards to a one-stop clinic after mentioning additional breast symptoms during the consultation. Symptoms included a swollen breast, nipple discharge and a lump. Patients who were deemed by telephone consultation to have a significant family history of breast or ovarian cancer were not redirected to a one-stop clinic but instead referred on to the family history team.

Women aged over 40 years (609/1,238; 49%) underwent bilateral mammography in keeping with local guidelines unless they had previously had mammography within 6 months of their telephone mastalgia clinic appointment. Of the women undergoing mammography, 25/609 (4%) were recalled for abnormalities on the imaging. Following full triple assessment these mammographically detected abnormalities were ultimately confirmed as normal glandular tissue (*n* = 2), pseudo-angiomatous stromal hyperplasia (*n* = 1), fibrocystic disease (*n* = 3), radial scar (*n* = 2), fibroadenomatoid change (*n* = 2), fibroadenoma (*n* = 2), cyst (*n* = 3), benign calcification (*n* = 3), ductal carcinoma in situ (DCIS) alone (*n* = 2/609, 0.4%) and invasive malignancy with or without DCIS (*n* = 5/609, 0.8%).

In all those assessed within the telephone mastalgia clinic, 7/1,238 (0.56%) women (or 1% [7/609] of those who had undergone a mammogram) were diagnosed with a breast malignancy (median age 72 years [range 55–81]). Five of these patients were in the age range to qualify for NHS breast screening. Further clinicopathological details of these seven patients are provided in Table 1.

**Table 1:** Clinicopathological details of seven women diagnosed with a breast malignancy from a cohort of 1,238 referred with a diagnosis of mastalgia alone and assessed in a telephone-based mastalgia clinic

Patient	Age (years)	Malignancy	Clinical finding (P score)*	Laterality of mastalgia	Laterality of breast malignancy
1	Over 70	7mm intermediate grade DCIS	No examination documented	Right	Left
2	Of screening age	9mm G2 IDC and 35mm HG DCIS	No examination documented	Right	Right
3	Of screening age	IG DCIS	P1	Bilateral	Left
4	Over 70	6mm IDC G2	P1	Left	Right
5	Over 70	12mm IDC G2	P1	Bilateral	Right
6	Over 70	27mm G2 IDC	P1	Right	Right
7	Over 70	IDC grade 2	P5	Left	Left

*P1 = clinically normal findings, P2 = clinically benign findings, P3 = clinically abnormal findings probably benign, P4 = clinical findings suspicious for breast cancer, P5 = clinical findings diagnostic of breast cancer.

DCIS = ductal carcinoma in situ; G2 = grade invasive carcinoma; HG = high grade DCIS; IDC = invasive ductal carcinoma; IG= intermediate grade DCIS

Because each patient was assessed face-to-face on recall, examination findings were also collated. These patients were seen by any clinician undertaking the clinic on that day and not necessarily by an ANP. Four of seven patients had a normal (P1) clinical examination despite the known abnormal finding on mammogram; for two there was no documentation. Mastalgia was not specific to the side of malignancy, being contralateral to that of the malignancy in two patients, ipsilateral in two, and two patients had bilateral mastalgia with a unilateral malignancy. No documentation on laterality was noted in one patient. One additional patient (aged 87 years) had been incorrectly triaged and was identified as having a malignant fixed mass to chest wall as identified on the referral letter. This patient was immediately referred into a breast cancer diagnostic clinic for further investigations.

Of the 1,238 patients assessed within the telephone clinic, 1,104 (89%) were identified as having a musculoskeletal aetiology for their symptoms. Only 39 women (3%) were diagnosed as having true mastalgia, i.e. pain arising from the breasts. Fifty women (4%) were identified as having pain caused by other conditions such as Raynaud’s phenomenon, cholecystitis and osteoarthritis. There was no documentation on 45 (4%) patients.

Data on re-referral of women to the breast clinic within 12 months of the telephone mastalgia appointment were available for 843 patients (68%). Of this cohort, 34 (4%) returned to our breast clinic within 12 months: 22 (2.6%) returning with the same symptom and 12 with new breast symptoms not reported previously. One patient (aged 61 years), initially assessed for left mastalgia, returned 10 months later with a new contralateral right breast lump, not previously reported, and was diagnosed with a lymph node negative, 8mm grade 1 invasive ductal/carcinoma. The initial mammogram performed as part of the telephone pain assessment clinic was normal bilaterally. The subsequent mammogram performed on representation 10 months later showed a 7mm indeterminate area of coarse calcification.

To assess patient satisfaction with the telephone mastalgia service, questionnaires were sent to 619 consecutive women. A response was received from 210 (34%) women of whom 195 (93%) expressed satisfaction with the consultation. Time saved (164; 82%) and convenience (131; 63%) were the most popular features of the service amongst responders. Almost all women (203; 97%) suggested they would recommend the service to family and friends.

## Discussion

The symptomatic breast two-week wait standard was introduced in the NHS in 2010 in order that all patients referred with breast symptoms would be seen within two weeks of referral, whether breast cancer was suspected or not. However, over time, the mismatch between capacity and demand has resulted in over half of patients referred on a two-week wait breast referral pathway waiting longer than the recommended time to be seen.^10^ This delay can negatively impact women with true “red flag” symptoms of breast cancer, e.g. a breast lump. Therefore, identifying a cohort of patients who can safely be managed outside the one-stop clinic would go some way to re-creating capacity for women with symptoms linked to breast cancer within breast outpatient services. Mastalgia accounts for approximately 20% of current breast clinic referrals in the UK, despite national guidance that it is best managed in primary care.^11^ Redirecting women with mastalgia out of a cancer diagnostic service could produce increased capacity for women with “red flag” cancer symptoms while simultaneously providing women with mastalgia a service specifically directed to addressing their needs for information and management of their pain symptoms. We have demonstrated an alternative management pathway for mastalgia patients uncoupled from the one-stop clinic. The service is telephone-based and ANP-led, founded on reproducible, multidisciplinary-developed, triage criteria. Our service has been shown to be associated with a high patient satisfaction rate. Over 97% of patients would recommend the service to others, with factors such as time saved and convenience being quoted by patients. We noted low rates (4%) of re-referral to breast clinic within 12 months, with almost all of these cases (97.4%) being for non-breast pain symptoms. The rate of cancer detection was low (0.6%) in breast pain patients, which is comparable with previously published data.^9,12,13^ All malignancies were diagnosed through mammography and this could well be considered opportunistic breast screening. Use of mammography in women with breast pain and an absence of any other breast symptoms is controversial. The Royal College of Radiologists state that breast pain alone is not an indication for imaging,^14^ and evidence as far back as 1998 suggests that imaging is only useful as a tool for reassurance.^15^ Despite the controversy, it remains common for many units to routinely offer mammography to women aged over 40 years with breast pain.^7–16^

The clinical effectiveness of routine mammography in women with breast pain and an absence of other breast symptoms requires further study.

In all, we show that four of every five women with mastalgia are able to avoid attending a one-stop clinic with a concomitant increase in capacity for women who have “red flag” symptoms concerning for a cancer diagnosis. Forty-nine per cent of patients in our study were referred for a mammogram, but these appointments were made outside the one-stop clinic environment and thus did not impact the assessment of women with red flag symptoms

Strengths of our study include prospective collection of data on a large number of women with mastalgia, with the vital element of obtaining patient feedback on their experience of using the service. The data from this study suggest that with a concise assessment tool and thorough questioning, the origin of the mastalgia and any further symptoms can be safely ascertained. It is, however, recognised that this assessment tool needs further validation, and there is further work currently ongoing. Weaknesses of the study include the fact that we are unable to guarantee that women originally assessed within our telephone clinic did not re-present to other breast clinics outside our region within the subsequent 12 months. However, as a unit with a large geographical footprint it was felt that presentation to other units in the area was unlikely. Also, a health economic assessment of the telephone service has not been conducted. This would be helpful in understanding the efficiency in economic terms of the telephone service.

Several studies have reported no association between presentation with breast pain and detection of breast cancer following clinical examination and imaging. Rates of breast cancer in women with mastalgia alone, in the absence of other breast symptoms, range from 0% to 0.4%.^17–19^ These data are similar to cancer detection rates in asymptomatic women within the NHS breast screening programme^20^ and suggest that any diagnosis of breast cancer accompanying the presence of mastalgia in a woman with no other breast symptoms is likely a coincidental occurrence.

Although women with mastalgia can be reassured that their symptoms do not suggest an underlying malignant diagnosis, they still deserve high-quality healthcare that addresses their concerns and offers evidence-based advice on management of their symptoms. It can be argued that referring women with mastalgia to cancer diagnostic clinics may offer a route for this reassurance and management. However, the effectiveness of this type of clinical environment in reducing concerns and anxieties has recently been challenged.^21,22^ Our telephone-based service relocates the care of these women out of a cancer diagnostic environment into a more appropriate care pathway designed to offer support and access to any necessary diagnostic facilities. The patient-level feedback on our service indicates that this is welcomed by women.

## Conclusions

We have presented an alternative pathway for the assessment of patients with mastalgia symptoms alone outside of a one-stop breast cancer diagnostic service via an ANP-led telephone clinic. This has proved to be safe and associated with high patient satisfaction levels. Such a development has resulted in almost 80% of women with mastalgia avoiding the need to attend one-stop diagnostic clinics, freeing up capacity for women with red flag breast cancer symptoms.

## Author contributions

KSE, CER and AG conceived the service and interpreted the results. KSE, AG and RF wrote the manuscript. KSE and CER collated the service data. HF planned and developed the patient-reported outcome measures.

## References

[1] Iddon J, Dixon JM. Mastalgia. *BMJ* 2013; **347**: f3288. 24336097 10.1136/bmj.f3288

[2] Dixon JM. *ABC of Breast Diseases*. 4th edn. Chichester: Blackwell; 2012; ix: 155 p.

[3] NHS England. *Hospital Episode Statistics 2019–2020*. https://digital.nhs.uk/data-and-information/publications/statistical/hospital-outpatient-activity/2019-2020 (cited March 2024).

[4] Hafiz SP, Barnes NLP, Kirwan CC. Clinical management of idiopathic mastalgia: a systematic review. *J Prim Health Care* 2018; **10**: 312–323. 31039960 10.1071/HC18026

[5] Sawyer L, Laking S, Gutteridge E. Relieving the pain of the new referral clinic. *Eur J Surg Oncol* 2018; **44**: 862–918.

[6] Joyce DP, Alamiri J, Lowery AJ *et al.* Breast clinic referrals: can mastalgia be managed in primary care? *Ir J Med Sci* 2014; **183**: 639–642. 24402166 10.1007/s11845-013-1066-z

[7] Sivarajah RW J, Mack J, Casas RS *et al.* A review of breast pain: causes, imaging recommendations, and treatment. *J Breast Imaging* 2020; **2**: 101–111. 38424883 10.1093/jbi/wbz082

[8] Mohallem Fonseca M, Lamb LR, Verma R *et al.* Breast pain and cancer: should we continue to work-up isolated breast pain? *Breast Cancer Res Treat* 2019; **177**: 619–627. 31309396 10.1007/s10549-019-05354-1

[9] Dave RV, Bromley H, Taxiarchi VP *et al.* No association between breast pain and breast cancer: a prospective cohort study of 10 830 symptomatic women presenting to a breast cancer diagnostic clinic. *Br J Gen Pract* 2022; **72**: e234–e243. 34990395 10.3399/BJGP.2021.0475 PMC8869188

[10] NHS England. *Provider based cancer waiting times for January 2021–22*. https://www.england.nhs.uk/statistics/statistical-work-areas/cancer-waiting-times/monthly-prov-cwt/2021-22-monthly-provider-cancer-waiting-times-statistics/provider-based-cancer-waiting-times-for-january-2021-22-final/ (cited March 2024).

[11] National Institute for Health & Care Excellence. *Suspected Cancer: Recognition and Referral (NG12)*. https://www.nice.org.uk/guidance/ng12 (cited March 2024).

[12] Locker AP, Manhire AR, Stickland V, *et al.* Mammography in symptomatic breast disease. *Lancet* 1989; **1**: 887–889. 2564959 10.1016/s0140-6736(89)92875-4

[13] Smallwood JA, Kye DA, Taylor I. Mastalgia; is this commonly associated with operable breast cancer? *Ann R Coll Surg Engl* 1986; **68**: 262–263. 3789622 PMC2498340

[14] The Royal College of Radiologists. *Guidance on Screening and Symptomatic Breast Imaging*. 4th edn. London: The Royal College of Radiologists; 2019.

[15] Duijm LE, Guit GL, Hendriks JH *et al.* Value of breast imaging in women with painful breasts: observational follow up study. *Br Med J* 1998; **317**: 1492–1495. 9831579 10.1136/bmj.317.7171.1492 PMC28731

[16] Arslan M, Küçükerdem HS, Can H, Tarcan E. Retrospective analysis of women with only mastalgia. *J Breast Health* 2016; **12**: 151. 28331753 10.5152/tjbh.2016.2944 PMC5351439

[17] Martin-Diaz M, Maes-Carballo M, Khan KS, Bueno-Cavanillas A. To image or not in noncyclic breast pain? A systematic review. *Curr Opin Obstet Gynecol* 2017; **29**: 404–412. 28961632 10.1097/GCO.0000000000000407

[18] Kushwaha AC, Shin K, Kalambo M *et al.* Overutilization of health care resources for breast pain. *AJR Am J Roentgenol* 2018; **211**: 217–223. 29792736 10.2214/AJR.17.18879

[19] Noroozian M, Stein LF, Gaetke-Udager K, Helvie MA. Long-term clinical outcomes in women with breast pain in the absence of additional clinical findings: mammography remains indicated. *Breast Cancer Res Treat* 2015; **149**: 417–424. 25556516 10.1007/s10549-014-3257-3

[20] NHS England & Association of Breast Surgery. An audit of screen detected breast cancers for the year of screening April 2019 to March 2020. https://associationofbreastsurgery.org.uk/media/359057/nhs-bsp-abs-audit-2019-20.pdf (cited March 2024).

[21] Howard MB, Battaglia T, Prout M, Freund K. The effect of imaging on the clinical management of breast pain. *J Gen Intern Med* 2012; **27**: 817–824. 22331398 10.1007/s11606-011-1982-4 PMC3378742

[22] Zarei F, Pishdad P, Hatami M, Zeinali-Rafsanjani B. Can breast ultrasound reduce patient's level of anxiety and pain? *Ultrasound* 2017; **25**: 92–97. 28567103 10.1177/1742271X17690021 PMC5438054

